# 3-[4-(Dimethyl­amino)­phen­yl]-1-(4a,8-dimethyl-1,2,3,4,4a,5,6,8a-octa­hydro­naphthalen-2-yl)prop-2-en-1-one

**DOI:** 10.1107/S160053681005333X

**Published:** 2010-12-24

**Authors:** Mohamed Tebbaa, Mohamed Akssira, Ahmed Elhakmaoui, Ahmed Benharref, Jean-Claude Daran, Moha Berraho

**Affiliations:** a Laboratoire de Chimie Bioorganique et Analytique, URAC 22 BP 146, FSTM, Université Hassan II, Mohammedia-Casablanca 20810 Mohammedia, Morocco; b Laboratoire de Chimie Biomoleculaire, Substances Naturelles et Réactivité, URAC16, Université Cadi Ayyad, Faculté des Sciences Semlalia, BP 2390, Bd My Abdellah, 40000 Marrakech, Morocco; c Laboratoire de Chimie de Coordination, 205 route de Narbonne, 31077 Toulouse Cedex 04, France

## Abstract

The title compound, C_23_H_31_NO, was semisynthesized from isocostic acid, isolated from the aerial part of *Inula Viscosa­* (L) Aiton [or *Dittrichia Viscosa­* (L) Greuter]. The cyclo­hexene ring has a half-chair conformation, whereas the cyclo­hexane ring displays a chair conformation. The dihedral angle between the latter ring and its substituent is 83.6 (7)°.

## Related literature

For background to the medicinal inter­est in *Inula Viscosa­* (*L*) Aiton [or *Dittrichia Viscosa­* (L) Greuter], see: Shtacher & Kasshman (1970 ▶); Bohlman & Gupta (1982 ▶); Azoulay *et al.* (1986 ▶); Bohlmann *et al.* (1977 ▶); Ceccherelli *et al.* (1988 ▶). For the synthesis, see: Kutney & Singh (1984 ▶). For conformational analysis, see: Cremer & Pople (1975 ▶).
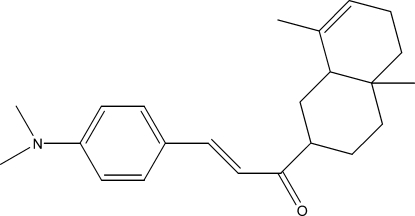


## Experimental

### 

#### Crystal data

C_23_H_31_NO
*M* *_r_* = 337.49
Monoclinic, 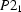

*a* = 6.0593 (4) Å
*b* = 7.2095 (7) Å
*c* = 21.8937 (19) Å
β = 91.860 (7)°
*V* = 955.91 (14) Å^3^
*Z* = 2
Mo *K*α radiation
μ = 0.07 mm^−1^
*T* = 298 K
0.6 × 0.25 × 0.10 mm

#### Data collection

Oxford Diffraction Xcalibur Eos Gemini ultra diffractometer
8394 measured reflections
2112 independent reflections
1894 reflections with *I* > 2σ(*I*)
*R*_int_ = 0.028

#### Refinement

*R*[*F* ^2^ > 2σ(*F* ^2^)] = 0.033
*wR*(*F* ^2^) = 0.089
*S* = 1.05
2112 reflections
230 parameters
1 restraint
H-atom parameters constrained
Δρ_max_ = 0.21 e Å^−3^
Δρ_min_ = −0.15 e Å^−3^

### 

Data collection: *CrysAlis PRO* (Oxford Diffraction, 2010 ▶); cell refinement: *CrysAlis PRO*; data reduction: *CrysAlis PRO*; program(s) used to solve structure: *SHELXS97* (Sheldrick, 2008 ▶); program(s) used to refine structure: *SHELXL97* (Sheldrick, 2008 ▶); molecular graphics: *ORTEP-3 for Windows* (Farrugia, 1997 ▶); software used to prepare material for publication: *WinGX* (Farrugia, 1999 ▶).

## Supplementary Material

Crystal structure: contains datablocks I, global. DOI: 10.1107/S160053681005333X/fj2378sup1.cif

Structure factors: contains datablocks I. DOI: 10.1107/S160053681005333X/fj2378Isup2.hkl

Additional supplementary materials:  crystallographic information; 3D view; checkCIF report

## Figures and Tables

**Fig. 1.**
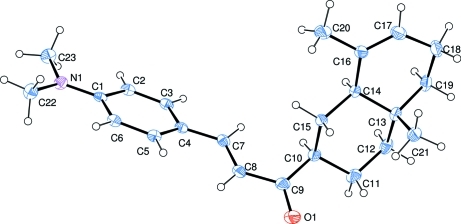
Molecular structure of the title compound with the atom-labelling scheme. Displacement ellipsoids are drawn at the 30% probability level. H atoms are represented as small spheres of arbitrary radii.

## supplementary crystallographic information

### Comment

Our work lies within the framework of the valorization of medicinals plants and concerning Inula Viscosa(*L*) Aiton or Dittrichia Viscosa (*L*) Greuter. This plant is widespread in Mediterranean area and extends to the Atlantic cost of Morocco It is a well known medicinal plant (Shtacher & Kasshman, 1970; Bohlman & Gupta, 1982) and has some pharmacological activities (Azoulay *et al.*, 1986). This plant has been the subject of chemical investigation in terms of isolating sesquiterpene lactones (Bohlmann *et al.*, 1977), sesquiterpene acids (Ceccherelli *et al.*, 1988). The isocostic acid is a major constituent of the dichloromethane extract of the Inula viscosa (*L*).The literature does not report any article on the transformation of this acid. In order to prepare products with high added value, we studied the reactivity of this acid. Thus, from this acid, we have prepared by reaction of Curtius the 1 - (4a, 8-dimethyl-1, 2,3,4,4 a, 5,6,8 a-octahydro- naphthalen-2-yl)-ethanone which was synthesized by Kutney *et al.*(1984). The Condensation of this ketone with 4-dimethylamino-benzaldehyde in the presence of sodium hydroxide allows us to obtain the title compound with a good yield of 80%. The structure of this new derivative of isocostic acid was established by NMR spectral analysis of 1H, 13 C and mass spectroscopy and confirmed by its single-crystal X-ray structure. The molecule is built up from two fused six-membered rings, substituted by 4-dimethyl-amino-phenylpropanoyl. The molecular structure of (I),Fig.1, shows the cyclohexene ring to adopt a half chair conformation as indicated by the total puckering amplitude Q(T)= 0.506 (2)Å and spherical polar angle θ = 48.8 (2)° with φ = 18.5 (3)°. By contrast the cyclohexane ring has a chair conformation with Q(T)= 0.574 (2)Å and spherical polar angle θ = 173.9 (2)° with φ = 28.6 (18)° (Cremer and Pople,1975).

### Experimental

In a flask was introduced a mixture of 500 mg (2.42 mmol), of 1 - (4a, 8-dimethyl-1, 2,3,4,4a,5,6,8 a-octahydro-naphthalen-2 -yl)-ethanone, 360 mg (2.42 mmol.) of 4-dimethylamino-benzaldehyde, 30 ml of anhydrous ethanol and 1 ml of a solution of sodium hydroxide(2 N). The mixture was stirred for three hours at room temperature. After neutralization followed by extraction three time with 20 ml of dichloromethane, the organic phase is dried over sodium sulfate, then evaporated under vacuum. Chromatography on a column of silica gel with hexane-ethyl acetate (98:2) as eluent of the residue allowed us to obtain 3-(4-dimethylamino-phenyl)(4a,8-dimethyl-1,2, 3,4,4a,5,6,8a-octahydro-naphthalene-2-yl)-propene-1-one with a yield of 80%. The title compound is recrystallized in hexane-ethyl acetate (95/5).

### Refinement

All H atoms were fixed geometrically and treated as riding with C—H = 0.96 Å (methyl),0.97 Å (methylene), 0.98Å (methine) with *U*_iso_(H) = 1.2Ueq(methylene, methine and OH) or *U*_iso_(H) = 1.5Ueq(methyl). In the absence of significant anomalous scattering, the absolute configuration could not be reliably determined and thus 1783 Friedel pairs were merged and any references to the Flack parameter were removed.

### Crystal data

**Table:**

C_23_H_31_NO	*F*(000) = 368
*M**_r_* = 337.49	*D*_x_ = 1.173 Mg m^−^^3^
Monoclinic, *P*2_1_	Mo *K*α radiation, λ = 0.71073 Å
Hall symbol: P 2yb	Cell parameters from 4058 reflections
*a* = 6.0593 (4) Å	θ = 3.0–28.5°
*b* = 7.2095 (7) Å	µ = 0.07 mm^−^^1^
*c* = 21.8937 (19) Å	*T* = 298 K
β = 91.860 (7)°	Prism, colourless
*V* = 955.91 (14) Å^3^	0.6 × 0.25 × 0.10 mm
*Z* = 2	

### Data collection

**Table:**

Oxford Diffraction Xcalibur Eos Gemini ultra diffractometer	1894 reflections with *I* > 2σ(*I*)
Radiation source: fine-focus sealed tube	*R*_int_ = 0.028
graphite	θ_max_ = 26.4°, θ_min_ = 3.0°
Detector resolution: 16.1978 pixels mm^-1^	*h* = −7→7
φ and ω scans	*k* = −9→7
8394 measured reflections	*l* = −27→27
2112 independent reflections	

### Refinement

**Table:**

Refinement on *F*^2^	Primary atom site location: structure-invariant direct methods
Least-squares matrix: full	Secondary atom site location: difference Fourier map
*R*[*F*^2^ > 2σ(*F*^2^)] = 0.033	Hydrogen site location: inferred from neighbouring sites
*wR*(*F*^2^) = 0.089	H-atom parameters constrained
*S* = 1.05	*w* = 1/[σ^2^(*F*_o_^2^) + (0.056*P*)^2^ + 0.0652*P*] where *P* = (*F*_o_^2^ + 2*F*_c_^2^)/3
2112 reflections	(Δ/σ)_max_ = 0.002
230 parameters	Δρ_max_ = 0.21 e Å^−^^3^
1 restraint	Δρ_min_ = −0.15 e Å^−^^3^

### Special details

**Table:**

Geometry. All s.u.'s (except the s.u. in the dihedral angle between two l.s. planes) are estimated using the full covariance matrix. The cell s.u.'s are taken into account individually in the estimation of s.u.'s in distances, angles and torsion angles; correlations between s.u.'s in cell parameters are only used when they are defined by crystal symmetry. An approximate (isotropic) treatment of cell s.u.'s is used for estimating s.u.'s involving l.s. planes.
Refinement. Refinement of *F*^2^ against ALL reflections. The weighted *R*-factor *wR* and goodness of fit *S* are based on *F*^2^, conventional *R*-factors *R* are based on *F*, with *F* set to zero for negative *F*^2^. The threshold expression of *F*^2^ > 2σ(*F*^2^) is used only for calculating *R*-factors(gt) *etc*. and is not relevant to the choice of reflections for refinement. *R*-factors based on *F*^2^ are statistically about twice as large as those based on *F*, and *R*- factors based on ALL data will be even larger.

### Fractional atomic coordinates and isotropic or equivalent isotropic displacement parameters (Å^2^)

**Table:**

	*x*	*y*	*z*	*U*_iso_*/*U*_eq_	
C1	0.7303 (3)	0.8137 (3)	1.01854 (8)	0.0250 (4)	
C2	0.9300 (3)	0.7287 (3)	1.00424 (8)	0.0278 (4)	
H2	1.0181	0.6768	1.0353	0.033*	
C3	0.9978 (3)	0.7208 (3)	0.94506 (8)	0.0280 (4)	
H3	1.1328	0.6656	0.9373	0.034*	
C4	0.8718 (3)	0.7923 (2)	0.89613 (8)	0.0263 (4)	
C5	0.6725 (3)	0.8750 (3)	0.91030 (8)	0.0287 (4)	
H5	0.5835	0.9236	0.8788	0.034*	
C6	0.6025 (3)	0.8873 (3)	0.96950 (8)	0.0281 (4)	
H6	0.4689	0.9450	0.9771	0.034*	
C7	0.9359 (3)	0.7711 (3)	0.83304 (8)	0.0295 (4)	
H7	0.8257	0.7832	0.8027	0.035*	
C8	1.1400 (3)	0.7357 (3)	0.81514 (9)	0.0345 (4)	
H8	1.2509	0.7321	0.8455	0.041*	
C9	1.2066 (3)	0.7020 (3)	0.75226 (9)	0.0371 (5)	
C10	1.0332 (3)	0.6566 (3)	0.70325 (8)	0.0321 (4)	
H10	0.9057	0.7376	0.7089	0.038*	
C11	1.1144 (4)	0.6858 (3)	0.63863 (9)	0.0422 (5)	
H11A	1.1362	0.8174	0.6317	0.051*	
H11B	1.2556	0.6244	0.6348	0.051*	
C12	0.9521 (4)	0.6105 (3)	0.59038 (8)	0.0407 (5)	
H12A	0.8182	0.6843	0.5905	0.049*	
H12B	1.0155	0.6235	0.5505	0.049*	
C13	0.8930 (3)	0.4083 (3)	0.60040 (8)	0.0299 (4)	
C14	0.7155 (3)	0.3441 (4)	0.55361 (8)	0.0396 (5)	
H14A	0.5853	0.4212	0.5571	0.048*	
H14B	0.7699	0.3597	0.5127	0.048*	
C15	0.6526 (3)	0.1432 (4)	0.56287 (9)	0.0458 (6)	
H15A	0.5140	0.1185	0.5410	0.055*	
H15B	0.7646	0.0643	0.5457	0.055*	
C16	0.6295 (3)	0.0958 (3)	0.62862 (9)	0.0408 (5)	
H16	0.5651	−0.0174	0.6379	0.049*	
C17	0.6948 (3)	0.2044 (3)	0.67505 (8)	0.0317 (4)	
C18	0.7946 (3)	0.3924 (3)	0.66407 (7)	0.0265 (4)	
H18	0.6723	0.4812	0.6646	0.032*	
C19	0.9605 (3)	0.4550 (3)	0.71385 (8)	0.0290 (4)	
H19A	1.0886	0.3744	0.7140	0.035*	
H19B	0.8940	0.4452	0.7534	0.035*	
C20	1.0979 (3)	0.2867 (3)	0.59410 (9)	0.0393 (5)	
H20A	1.0624	0.1607	0.6040	0.059*	
H20B	1.2134	0.3303	0.6215	0.059*	
H20C	1.1466	0.2929	0.5529	0.059*	
C21	0.6499 (4)	0.1524 (4)	0.73956 (10)	0.0524 (6)	
H21A	0.5633	0.0409	0.7399	0.079*	
H21B	0.5703	0.2508	0.7586	0.079*	
H21C	0.7871	0.1321	0.7617	0.079*	
C22	0.8030 (3)	0.7487 (3)	1.12674 (9)	0.0393 (5)	
H22A	0.8180	0.6174	1.1209	0.059*	
H22B	0.7360	0.7719	1.1651	0.059*	
H22C	0.9462	0.8058	1.1267	0.059*	
C23	0.4516 (3)	0.8908 (3)	1.09306 (9)	0.0374 (5)	
H23A	0.3430	0.7989	1.0819	0.056*	
H23B	0.4199	1.0037	1.0712	0.056*	
H23C	0.4482	0.9137	1.1362	0.056*	
N1	0.6663 (2)	0.8252 (2)	1.07791 (7)	0.0325 (4)	
O1	1.4040 (2)	0.6982 (3)	0.74180 (8)	0.0544 (5)	

### Atomic displacement parameters (Å^2^)

**Table:**

	*U*^11^	*U*^22^	*U*^33^	*U*^12^	*U*^13^	*U*^23^
C1	0.0224 (7)	0.0172 (9)	0.0354 (8)	−0.0036 (7)	−0.0016 (6)	−0.0015 (7)
C2	0.0252 (8)	0.0228 (10)	0.0352 (9)	0.0016 (8)	−0.0042 (7)	0.0000 (8)
C3	0.0235 (7)	0.0214 (10)	0.0389 (9)	0.0023 (7)	0.0001 (7)	−0.0031 (8)
C4	0.0273 (8)	0.0171 (10)	0.0342 (9)	−0.0033 (7)	−0.0014 (7)	−0.0016 (7)
C5	0.0267 (8)	0.0229 (10)	0.0361 (9)	−0.0011 (7)	−0.0059 (7)	0.0015 (8)
C6	0.0230 (7)	0.0206 (9)	0.0406 (10)	0.0026 (7)	−0.0027 (7)	−0.0002 (8)
C7	0.0353 (9)	0.0189 (10)	0.0339 (9)	−0.0044 (7)	−0.0025 (7)	−0.0009 (7)
C8	0.0334 (9)	0.0317 (11)	0.0382 (10)	−0.0077 (9)	−0.0016 (7)	−0.0066 (8)
C9	0.0374 (9)	0.0279 (11)	0.0463 (11)	−0.0089 (9)	0.0073 (8)	−0.0057 (9)
C10	0.0379 (9)	0.0269 (11)	0.0318 (9)	−0.0051 (8)	0.0072 (7)	−0.0005 (8)
C11	0.0544 (12)	0.0335 (12)	0.0396 (10)	−0.0104 (10)	0.0152 (9)	0.0056 (9)
C12	0.0547 (11)	0.0415 (13)	0.0267 (9)	0.0020 (10)	0.0115 (8)	0.0086 (9)
C13	0.0314 (8)	0.0349 (11)	0.0237 (8)	0.0046 (8)	0.0054 (7)	−0.0005 (8)
C14	0.0383 (10)	0.0566 (15)	0.0239 (8)	0.0093 (10)	0.0009 (7)	−0.0053 (9)
C15	0.0403 (11)	0.0575 (15)	0.0395 (10)	−0.0011 (11)	−0.0006 (8)	−0.0215 (10)
C16	0.0335 (9)	0.0391 (13)	0.0499 (11)	−0.0039 (9)	0.0004 (8)	−0.0091 (10)
C17	0.0278 (8)	0.0328 (11)	0.0346 (9)	−0.0028 (8)	0.0021 (7)	−0.0024 (8)
C18	0.0271 (8)	0.0292 (10)	0.0233 (8)	0.0029 (8)	0.0036 (6)	−0.0010 (7)
C19	0.0349 (9)	0.0278 (10)	0.0244 (8)	−0.0059 (8)	0.0027 (7)	0.0008 (7)
C20	0.0306 (9)	0.0467 (14)	0.0410 (10)	0.0038 (9)	0.0069 (8)	−0.0074 (9)
C21	0.0661 (14)	0.0480 (15)	0.0433 (11)	−0.0277 (13)	0.0041 (10)	0.0073 (11)
C22	0.0369 (9)	0.0459 (14)	0.0350 (9)	0.0075 (10)	0.0017 (7)	0.0030 (9)
C23	0.0329 (9)	0.0344 (11)	0.0452 (11)	0.0081 (9)	0.0050 (8)	−0.0013 (9)
N1	0.0264 (7)	0.0366 (10)	0.0347 (8)	0.0037 (7)	0.0020 (6)	0.0032 (7)
O1	0.0352 (7)	0.0667 (12)	0.0619 (9)	−0.0137 (8)	0.0125 (7)	−0.0192 (9)

### Geometric parameters (Å, °)

**Table:**

C1—N1	1.371 (2)	C13—C18	1.538 (2)
C1—C2	1.401 (2)	C14—C15	1.513 (4)
C1—C6	1.407 (2)	C14—H14A	0.9700
C2—C3	1.373 (2)	C14—H14B	0.9700
C2—H2	0.9300	C15—C16	1.490 (3)
C3—C4	1.394 (2)	C15—H15A	0.9700
C3—H3	0.9300	C15—H15B	0.9700
C4—C5	1.390 (2)	C16—C17	1.333 (3)
C4—C7	1.455 (2)	C16—H16	0.9300
C5—C6	1.380 (2)	C17—C21	1.495 (3)
C5—H5	0.9300	C17—C18	1.507 (3)
C6—H6	0.9300	C18—C19	1.527 (2)
C7—C8	1.334 (3)	C18—H18	0.9800
C7—H7	0.9300	C19—H19A	0.9700
C8—C9	1.467 (3)	C19—H19B	0.9700
C8—H8	0.9300	C20—H20A	0.9600
C9—O1	1.226 (2)	C20—H20B	0.9600
C9—C10	1.513 (3)	C20—H20C	0.9600
C10—C11	1.527 (2)	C21—H21A	0.9600
C10—C19	1.538 (3)	C21—H21B	0.9600
C10—H10	0.9800	C21—H21C	0.9600
C11—C12	1.520 (3)	C22—N1	1.441 (2)
C11—H11A	0.9700	C22—H22A	0.9600
C11—H11B	0.9700	C22—H22B	0.9600
C12—C13	1.519 (3)	C22—H22C	0.9600
C12—H12A	0.9700	C23—N1	1.433 (2)
C12—H12B	0.9700	C23—H23A	0.9600
C13—C20	1.530 (3)	C23—H23B	0.9600
C13—C14	1.533 (2)	C23—H23C	0.9600
			
N1—C1—C2	120.75 (15)	C15—C14—H14B	109.2
N1—C1—C6	122.33 (15)	C13—C14—H14B	109.2
C2—C1—C6	116.91 (15)	H14A—C14—H14B	107.9
C3—C2—C1	121.08 (16)	C16—C15—C14	112.40 (17)
C3—C2—H2	119.5	C16—C15—H15A	109.1
C1—C2—H2	119.5	C14—C15—H15A	109.1
C2—C3—C4	122.42 (16)	C16—C15—H15B	109.1
C2—C3—H3	118.8	C14—C15—H15B	109.1
C4—C3—H3	118.8	H15A—C15—H15B	107.9
C5—C4—C3	116.43 (16)	C17—C16—C15	124.5 (2)
C5—C4—C7	121.17 (15)	C17—C16—H16	117.7
C3—C4—C7	122.26 (16)	C15—C16—H16	117.7
C6—C5—C4	122.21 (16)	C16—C17—C21	120.97 (19)
C6—C5—H5	118.9	C16—C17—C18	121.17 (17)
C4—C5—H5	118.9	C21—C17—C18	117.56 (17)
C5—C6—C1	120.93 (16)	C17—C18—C19	114.25 (15)
C5—C6—H6	119.5	C17—C18—C13	112.40 (15)
C1—C6—H6	119.5	C19—C18—C13	111.05 (13)
C8—C7—C4	125.31 (16)	C17—C18—H18	106.2
C8—C7—H7	117.3	C19—C18—H18	106.2
C4—C7—H7	117.3	C13—C18—H18	106.2
C7—C8—C9	126.40 (17)	C18—C19—C10	110.91 (16)
C7—C8—H8	116.8	C18—C19—H19A	109.5
C9—C8—H8	116.8	C10—C19—H19A	109.5
O1—C9—C8	118.61 (18)	C18—C19—H19B	109.5
O1—C9—C10	121.48 (17)	C10—C19—H19B	109.5
C8—C9—C10	119.69 (16)	H19A—C19—H19B	108.0
C9—C10—C11	112.96 (16)	C13—C20—H20A	109.5
C9—C10—C19	107.05 (17)	C13—C20—H20B	109.5
C11—C10—C19	111.88 (16)	H20A—C20—H20B	109.5
C9—C10—H10	108.3	C13—C20—H20C	109.5
C11—C10—H10	108.3	H20A—C20—H20C	109.5
C19—C10—H10	108.3	H20B—C20—H20C	109.5
C12—C11—C10	111.96 (16)	C17—C21—H21A	109.5
C12—C11—H11A	109.2	C17—C21—H21B	109.5
C10—C11—H11A	109.2	H21A—C21—H21B	109.5
C12—C11—H11B	109.2	C17—C21—H21C	109.5
C10—C11—H11B	109.2	H21A—C21—H21C	109.5
H11A—C11—H11B	107.9	H21B—C21—H21C	109.5
C13—C12—C11	113.12 (17)	N1—C22—H22A	109.5
C13—C12—H12A	109.0	N1—C22—H22B	109.5
C11—C12—H12A	109.0	H22A—C22—H22B	109.5
C13—C12—H12B	109.0	N1—C22—H22C	109.5
C11—C12—H12B	109.0	H22A—C22—H22C	109.5
H12A—C12—H12B	107.8	H22B—C22—H22C	109.5
C12—C13—C20	109.95 (17)	N1—C23—H23A	109.5
C12—C13—C14	110.92 (17)	N1—C23—H23B	109.5
C20—C13—C14	108.71 (16)	H23A—C23—H23B	109.5
C12—C13—C18	107.62 (15)	N1—C23—H23C	109.5
C20—C13—C18	112.25 (15)	H23A—C23—H23C	109.5
C14—C13—C18	107.38 (14)	H23B—C23—H23C	109.5
C15—C14—C13	111.95 (18)	C1—N1—C23	121.75 (15)
C15—C14—H14A	109.2	C1—N1—C22	120.44 (15)
C13—C14—H14A	109.2	C23—N1—C22	117.33 (15)
